# Metabolism, Morphology and Function of the Anterior Abdominal Wall Musculature in Ventral Hernias and After Repair: A Narrative Review and Research Agenda

**DOI:** 10.3390/jpm16080419

**Published:** 2026-08-06

**Authors:** Sergey Yu. Muraviev, Zakhar A. Akulov, Maria A. Sukhanova, Miroslava O. Pilipenko, Evgeniy A. Tarabrin, Zelimkhan G. M. Berikkhanov, Milena Yu. Ivanova, Andrey M. Nikolaev, Vadim S. Razumovsky, Vladislav S. Rakintsev, Aleksey G. Kotelnikov, Sara Nourmahal, Alexey L. Shestakov

**Affiliations:** Sechenov First Moscow State Medical University (Sechenov University), 119048 Moscow, Russia

**Keywords:** ventral hernia, myosteatosis, sarcopenia, metabolic syndrome, abdominal wall reconstruction, transversus abdominis release

## Abstract

**Objectives:** To assess whether muscle morphology and metabolism can inform personalised care in ventral hernias and to define which parts of a proposed postoperative remodelling model are supported by direct evidence. **Methods:** We conducted a structured narrative review of PubMed/MEDLINE, Scopus and Web of Science from 1 January 2010 through 30 April 2026. Two authors selected 38 publications. Evidence was classified as direct when generated in ventral or incisional hernia cohorts and indirect when drawn from inguinal hernia, transplantation, geriatric, oncological, animal or general muscle studies. No quantitative synthesis was undertaken. **Results:** Direct studies document lateral muscle displacement, impaired abdominal wall function, inconsistent associations between low muscle mass and clinical outcomes, and CT-derived increases in muscle cross-sectional areas after transversus abdominis release (TAR). The evidence for anterior abdominal wall myosteatosis, biomarker-guided care and the three proposed remodelling trajectories remains largely indirect. In a 37-patient TAR series, the median rectus abdominis cross-sectional area increased by 16.1% (IQR 11.4–27.0%); muscle function and metabolic recovery were not measured. **Conclusions:** Routine CT can yield muscle area, attenuation and intermuscular adipose tissue measures without further imaging. No anterior abdominal wall-specific thresholds or biomarker cut-offs have been validated, so these measures should not determine current treatment in isolation. Prospective studies should test whether combined imaging, functional and clinical phenotyping improves the selection of prehabilitation and follow-up.

## 1. Introduction

Incisional ventral hernias develop in 10–20% of patients after midline laparotomy and may reach 30% in high-risk groups [1]. At five years, the reported recurrence rate approaches 32% after mesh-augmented repair and 63% after primary non-mesh closure [2]. These estimates span different eras and techniques, but they indicate that operative method and prosthetic material do not account for all outcome variation.

Preoperative assessment usually centres on defect geometry, loss of domain and the feasibility of fascial closure. Muscle area and quality are not routinely measured. Obesity and components of metabolic syndrome often coexist with a ventral hernia, but their combined effect on AAW muscle has not been quantified. The prevalence of myosteatosis, sarcopenia and their overlap in this population is also unknown.

The published studies address abdominal wall atrophy, fibrosis, proteolysis and postoperative morphometry separately. They do not establish whether a preoperative muscle phenotype predicts functional recovery, complications or recurrence. The present review therefore separates observed findings from the mechanisms and trajectories proposed to connect them.

This review addresses three questions: how obesity, metabolic syndrome and hernia carriage may relate to anterior abdominal wall (AAW) phenotype; what is known about muscle change after repair; and whether imaging, functional testing or biomarkers could support personalised management. A database search was used to locate and map the literature for a structured narrative synthesis. The evidence from ventral or incisional hernia cohorts is treated as direct. The evidence from inguinal hernia, transplantation, geriatric or oncological cohorts, animal models and general muscle biology is labelled indirect. The three postoperative trajectories in Section 3.4 are hypotheses for prospective testing.

## 2. Materials and Methods

PubMed/MEDLINE, Scopus and Web of Science were searched from 1 January 2010 through 30 April 2026; the final search was run on 30 April 2026. The English-language filter was applied. Search strings combined four concept blocks: (A) ventral hernia, incisional hernia, abdominal wall or abdominal wall reconstruction; (B) sarcopenia, myosteatosis, muscle atrophy, muscle fibrosis or muscle remodelling; (C) obesity, metabolic syndrome, collagen metabolism, matrix metalloproteinase or reperfusion injury; and (D) botulinum toxin, component separation, transversus abdominis release, GLP-1 receptor agonist or neuromuscular electrical stimulation. Terms within each block were combined with OR; block A was then intersected (AND) with the union of blocks B, C and D. Syntax was adapted to each database; the complete strings are provided in Supplementary Table S1. Reference lists were hand-searched. No date exception was applied; all included sources fall within this window.

Titles and abstracts were screened independently by two authors; potentially eligible full texts were then assessed against the inclusion criteria by the same two authors, and a third author verified the selection and resolved disagreements. The review retained systematic reviews and meta-analyses; cohort studies; imaging or histological series with quantitative measures; and animal experiments when no human study addressed the mechanism. Case reports, editorials and studies without a hernia, AAW muscle, extracellular-matrix or relevant intervention outcome were excluded. A formal record of retrieved, duplicate and excluded records was not preserved; numerical study flow and a PRISMA diagram therefore cannot be reported. This omission is stated in the limitations.

For synthesis, each source was classified by population and inferential distance. Ventral or incisional hernia studies were direct. Inguinal hernia tissue studies were direct for hernia biology but indirect for ventral AAW muscle; transplantation, geriatric, oncological and general muscle studies were indirect; animal studies were preclinical. We extracted each study’s design, population, anatomical site, measurements and outcomes. Thirty-eight publications were retained. No protocol was registered, no pooled effect was calculated, and no formal risk-of-bias instrument was applied. The structured search and the direct/indirect classification improve traceability, while study selection and synthesis remain narrative.

## 3. Results

### 3.1. The Hernia-Carrier Triad: Obesity, Metabolic Syndrome and Hernia

Obesity (body mass index, BMI, above 30 kg/m^2^) raises the risk of both primary and postoperative hernias with a relative risk of 1.34–2.08 [3]. Visceral adiposity creates a chronic elevation of intra-abdominal pressure (IAP) directed at the linea alba; the strength of the linea alba depends on collagen quality [4]. In an obese patient, both parameters are likely altered simultaneously: pressure is elevated and collagen quality is impaired.

Obesity and metabolic comorbidity are common in patients considered for ventral-hernia repair, although their prevalence and duration are not reported uniformly. The abdominal wall may therefore have been exposed to altered loading and adipose-related inflammation before repair. BMI and defect dimensions are recorded routinely; muscle tissue quality usually is not.

Targeted epidemiological data on metabolic syndromes in ventral hernia patients are sparse. Mechanistic links are inferred from general muscle and adipose biology rather than demonstrated in the AAW. Chronic hyperinsulinaemia can disrupt the IGF-1/PI3K/Akt signalling involved in muscle protein balance [5]. Hypertrophied visceral adipocytes release TNF-α, IL-6 and TGF-β [6]; these mediators can promote proteolysis, catabolism and fibrosis in other settings. Their activity and clinical effect in hernia-carrier AAW muscles have not been measured directly.

The available literature suggests that low muscle attenuation or excess intermuscular fat can overlap with low muscle area. It does not establish a two-pathway natural history in the AAW.

Myosteatosis (myofatty dystrophy) develops with visceral obesity and an excess of free fatty acids. Lipid stores accumulate in the inter-muscular space and within myocytes. Fibres preserve their cellular structure but operate under what may be termed metabolic poisoning: insulin sensitivity is reduced and mitochondrial function is impaired [5]. On a CT scan, myosteatosis manifests as a reduction of the radiographic density of the muscle (measured in Hounsfield units, HU). In a prospective cohort of older inpatients [7], muscle quality indicators derived from chest CT scans at the T12 level were independently associated with in-hospital death after adjustments for sex and other confounders: each additional 1 cm^2^ of intermuscular adipose tissue increased the odds of death (OR 1.09; 95% CI 1.05–1.14), whereas each additional Hounsfield unit of skeletal muscle radiodensity reduced them (OR 0.93 per 1-HU increase; 95% CI 0.90–0.96). Expressed in the direction relevant to myosteatosis, each 1-HU decrement corresponds to approximately 8% higher odds of in-hospital death (inverted OR 1.08; 95% CI 1.04–1.11). These estimates derive from a geriatric acute-care population measured at a thoracic landmark; their transfer to the anterior abdominal wall is an assumption, not a finding.

Sarcopenia presents the opposite picture: reduced muscle mass, without obligatory fat infiltration. It develops with prolonged catabolism (diabetes mellitus, malignancy, severe malnutrition) or with age-related involution; denervation with insufficient reinnervation and myostatin-driven atrophic programs are central [8]. A systematic review of body-composition analyses in abdominal wall hernia repairs [9] retrieved 201 records and included four cohort studies; no randomised trial was found, and no quantitative synthesis was performed. The included studies differed considerably in the axial landmark and the muscle groups analysed and in how sarcopenia was defined. Two of the four reported an association between low skeletal muscle mass and adverse outcomes—hernia recurrence, postoperative ileus and prolonged hospitalisation; the remaining two did not. Direct evidence in the hernia population therefore exists, but it is confined to indices of muscle quantity, it is heterogeneous, and it is internally inconsistent. A retrospective CT analysis of 667 kidney recipients [10] did not detect a statistically significant influence of myosteatosis or sarcopenia on hernia risk (*p* > 0.05), although the limited size of the imaging subgroup and methodological heterogeneity leave the negative finding open to interpretation.

The two phenotypes may coexist in the same patient. Sarcopenic obesity, in which reduced muscle mass coincides with excess adipose tissue, combines features of both states and may create the least favourable conditions for surgery: mechanical weakness of the wall together with a metabolic incapacity to respond appropriately to restored loading. Population estimates of sarcopenic obesity in hernia carriers are absent, although the association of sarcopenia with adverse outcomes has been documented in several cohorts of parastomal hernias [11].

Muscle phenotype may influence rehabilitation potential, but categorical claims about reversibility are not supported in hernia cohorts. Non-hernia studies suggest that muscle attenuation and intermuscular fat can change with weight loss and loading; chronic lipotoxicity and fibrosis may limit recovery [5]. Advanced sarcopenia may also respond to resistance exercise and nutrition, although restored muscle volume does not prove recovered function. Table 1 therefore describes candidate phenotypes and their evidence status without assigning a fixed postoperative trajectory.

### 3.2. Morphological Changes in Chronic Hernias

When the midline aponeurosis loses continuity, the external oblique, internal oblique and transversus abdominis lose their medial fixation [12,13]. Lateral displacement and altered muscle dimensions have been documented on imaging, although studies differ in technique and measurement level [12,13,14]. In a rat model of a chronic incisional hernia, the internal-oblique fibre cross-sectional area fell from 2669 to 1792 µm^2^ (*p* < 0.001); mesh repair partly reversed atrophy and the shift towards type IIa fibres [15]. These experimental findings cannot be assumed to occur to the same degree in humans.

Human evidence is less specific. Imaging and functional reviews report altered muscle position, thickness and stiffness, but human biopsy data are scarce [12,16,17]. Previous surgery can injure segmental neurovascular bundles and produce denervation atrophy or a pseudohernia [12]. Fibre-type change is therefore supported mainly by animal work, whereas displacement and functional impairment have direct human evidence. The biomechanical implications for mesh placement and repair geometry have been reviewed separately [18].

Here, morphology distinguishes the two phenotypes. With sarcopenia, the dominant features are fibre atrophy and replacement by fibrous tissue. With myosteatosis, lipid inclusions accumulate in the inter-muscular space and within fibres while cell size is relatively preserved. CT densitometry can in principle separate them: a reduction of muscle radiographic density argues for fatty infiltration; a normal or elevated density with a reduced volume argues for fibrotic atrophy. No standardised protocol of such an assessment for AAW musculature before hernia repair has been developed.

Denervation may limit recovery after reconstruction. Motor innervation of the abdominal muscles is segmental, through the intercostal nerves (Th7–Th11), subcostal nerve (Th12) and L1 branches in the plane between the internal oblique and transversus abdominis [12]. Lateral, subcostal or lumbar incisions can injure these bundles. Prolonged denervation is associated with fibro-fatty replacement, but no duration threshold for recovery of AAW muscle after hernia repair has been defined [12,19]. An increase in the CT area may represent contractile, fibrotic or fatty tissue; the CT area alone cannot distinguish them.

Defect width may modify muscle remodelling, but no validated threshold separates reversible from fixed changes. The 10 cm cut-off used in some clinical planning reflects anatomical difficulty and lower closure rates without adjuncts [20,21], not a proven biological point of no return. Prospective imaging and biopsy studies stratified by both defect width and hernia duration are needed.

### 3.3. The Metabolic Profile of Muscles in Hernias

Evidence for increased proteolytic activity comes mainly from fascia and inguinal hernias rather than ventral AAW muscle. A systematic review of nine inguinal-hernia studies found MMP-2 to be the most consistently altered metalloproteinase, with variable results for other MMPs [22]. In 40 men with inguinal hernias, MMP-2 rose with age (r = 0.537; *p* < 0.001) while TIMP-2 fell (r = −0.759; *p* < 0.001) [23]. These findings concern inguinal fascia and circulating markers; their relevance to ventral AAW muscle remains indirect.

Further indirect evidence comes from a prospective cohort of 392 patients after a subcostal laparotomy [4]. A transversalis-fascia collagen measure, involving a picrosirius red-stained fibre area, was associated with major complications: 6 of 125 patients above a threshold of 0.771 developed Clavien–Dindo grade III or higher complications, compared with 25 of 134 who were below it (*p* < 0.01; AUC 0.606). The modest discrimination and non-hernia population preclude use of this threshold for ventral-hernia decisions.

The MMP/TIMP balance is a mechanistic candidate, but no circulating or tissue ratio has been validated as a measure of ventral AAW muscle status. P1NP, Pro-C3 and Raman spectroscopy are also investigational [1]. These measures may be suitable endpoints in prospective studies; none of these currently have a cut-off that can select an operation, prehabilitation regimen or rehabilitation schedule. Table 2 groups the candidate markers by source and evidentiary status.

In general muscle and adipose studies, TNF-α, IL-6 and TGF-β are linked to reactive oxygen species, metalloproteinase signalling and altered collagen remodelling [1,5,6]. Whether this sequence occurs in ventral AAW muscle, and whether it changes scar strength after repair, has not been tested.

TGF-β is one candidate link between mechanical stretch, inflammation and fibrosis [5]. Both stimuli may coexist in obesity, but their combined effect has not been measured in ventral AAW muscle.

A humanised aromatase mouse model provides preclinical evidence for an ESR1-dependent profibrotic programme in lower-abdominal fibroblasts [24]. Fibroblast-specific ESR1 ablation and fulvestrant reduced fibrosis and herniation in that model. Human ventral-hernia data are absent, so this finding supports mechanistic study rather than a therapeutic recommendation.

### 3.4. Pathogenetic Chain: From Obesity to Surgery and Back

**Figure 1:**
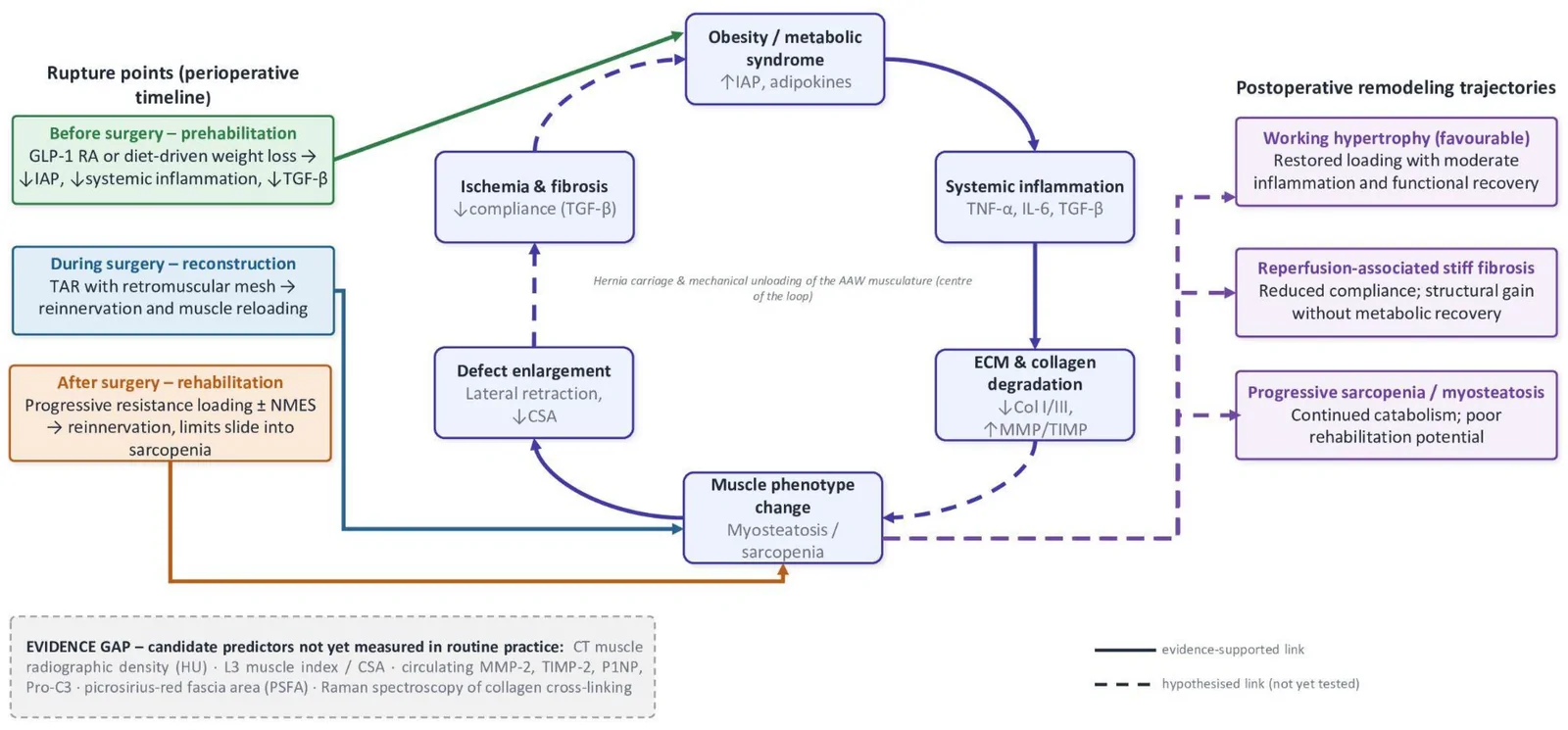
Metabolic–morphological vicious cycle in ventral hernias and three rupture points across the perioperative timeline. The central loop summarises the hypothesised reinforcing relationships between obesity/metabolic syndrome, systemic inflammation, the extracellular matrix and collagen degradation, muscle phenotype change (myosteatosis or sarcopenia), hernia enlargement, and ischaemia/fibrosis. The solid arrows represent links supported by direct or indirect evidence in hernia studies (e.g., MMP/TIMP imbalance, lateral muscle retraction, CSA loss); the dashed arrows mark hypothesised links extrapolated from non-hernia muscle biology that have not yet been prospectively verified in ventral hernia populations. The three lateral panels (green, blue, orange) denote the perioperative time windows during which the cycle can be ruptured by, respectively, metabolic and biomechanical prehabilitation, structural reconstruction with muscle reloading, and progressive rehabilitation. The right-hand panel depicts three competing postoperative remodelling trajectories whose probability is hypothesised to depend on baseline muscle phenotype. The bottom panel lists candidate predictors of trajectory that are currently not measured in routine practice; grey shading marks the corresponding evidence gap.

Visceral adiposity can raise intra-abdominal pressure. Loss of medial fixation alters AAW loading [12], while obesity-related inflammation can influence the extracellular matrix and muscle metabolism [5,6]. These processes may coexist, but no longitudinal hernia study has shown that they form the causal sequence depicted in Figure 1.

Pain and activity limitation may promote deconditioning and a loss of muscle reserve. A feedback effect from hernia to obesity or sarcopenia is biologically plausible, but it has not been measured directly in a longitudinal hernia cohort.

Hernia duration may represent cumulative exposure to altered loading and reduced activity, but no study validates a duration–BMI combination as a prognostic stage. Short and long durations, obesity class and diabetes should be recorded as continuous or prespecified clinical variables rather than used as biological cut-offs.

Repair changes abdominal-wall geometry and loading. We use three possible trajectories to organise testable mechanisms: fibrotic remodelling after an exaggerated inflammatory response; morphometric recovery with preserved function; and persistent or progressive muscle loss. No prospective cohort has assigned patients to these trajectories.

Trajectory A is a proposed profibrotic response in patients with impaired perfusion or marked metabolic disease. The evidence for ischaemia-reperfusion signalling and TGF-β-mediated fibrosis comes from general muscle biology [5]; a classical reperfusion syndrome has not been demonstrated after ventral hernia repair. The proposed link to a stiff, poorly functioning reconstructed wall therefore remains untested.

Trajectory B is morphometric recovery after restoration of loading. In 37 patients after open TAR, the median CT cross-sectional area increased by 16.1% (IQR 11.4–27.0%) in the rectus abdominis, 16.5% (IQR 8.5–20.1%) in the internal oblique and 9.5% (IQR 6.5–12.7%) in the external oblique (all *p* < 0.001) [14]. Function, muscle attenuation and metabolic markers were not measured; the CT expansion should not be labelled hypertrophy or metabolic recovery.

Trajectory C is a persistent loss of muscle mass or quality in the presence of low baseline reserve, denervation or ongoing catabolism. Resistance loading can support reinnervation in other settings [8], but no trial has shown that it changes a post-repair AAW trajectory or recurrence risk.

Baseline muscle state is one candidate determinant among operative technique, defect geometry, complications, nutrition and postoperative activity. No validated model predicts an individual trajectory.

### 3.5. Functional Disturbances and Postoperative Dynamics

A systematic review of 27 studies on AAW function found that isometric or isokinetic dynamometry was used in 88.9% [17]. In patients with an incisional hernia wider than 4 cm, peak trunk-flexor force was about 20% lower than in the healthy controls, and force development differed by 168–290 N·s^−1^ (*p* < 0.003) [17]. Each additional centimetre of defect width was associated with an approximately 5 N lower peak force [16]. Reference [25] reviews how functional assessment is used in contemporary abdominal wall reconstruction.

Some surface electromyography studies report increased activity despite muscle thinning [12,17], a finding interpreted as compensatory motor-unit recruitment. Surface electromyography alone does not establish the proposed links to fatigue, back pain or diaphragmatic dysfunction.

A meta-analysis of 163 patients found fewer wound complications after endoscopic rather than open anterior component separation (OR 0.27; 95% CI 0.12–0.58; *p* < 0.001), with no clear difference in recurrence (13% vs. 16%; OR 0.76; 95% CI 0.29–1.98) [26]. A meta-analysis of an open TAR series reported recurrence rates of 3.0–5.5%, compared with 11.9% after open anterior separation [27]. Preservation of neurovascular pedicles is a plausible explanation for some differences [13,26], but these comparisons do not establish a muscle phenotype–treatment interaction.

### 3.6. Prehabilitation: Botulinum Toxin, GLP-1 Receptor Agonists and Neuromuscular Electrical Stimulation

Botulinum toxin type A (BTA) produces a measurable anatomical effect before complex reconstruction. Meta-analyses report lateral-muscle elongation of approximately 3.2–4.0 cm per side and a higher probability of fascial closure [20,21,28,29]. BTA combined with progressive pneumoperitoneum was associated with a pooled closure proportion of 0.94 (95% CI 0.89–0.98) and recurrence proportion of 0.03 (95% CI 0.01–0.06) [30,31]. A propensity-weighted cohort reported lower recurrence [30], while patient-reported data remain heterogeneous [32]. These studies address anatomy and clinical outcomes, not metabolic muscle phenotype.

GLP-1 receptor agonists address obesity and metabolic risk. In a retrospective series of 70 patients awaiting hernia repair, the mean BMI fell by 5.3 ± 3.5 kg/m^2^ over 8.2 ± 4.9 months; recurrence was 3.0% after a median 5.9 months [33]. The uncontrolled design, short follow-up and absence of muscle measurements limit inferences. The study supports the feasibility of medical weight optimisation, not a phenotype-specific effect on AAW remodelling.

The evidence for neuromuscular electrical stimulation (NMES) is indirect. A meta-analysis of exercise, nutrition and NMES for sarcopenic obesity included 29 trials, six of which assessed NMES. NMES increased skeletal muscle index by 0.26 kg/m^2^ (95% CI 0.22–0.29) and reduced body-fat percentage by 2.01 points (95% CI 0.48–3.54), with I^2^ = 93% for fat percentage [34]. A separate review of 18 trials after thoracic or abdominal surgery found insufficient evidence for abdominal-surgery populations [35]. No trial has tested NMES of the AAW in ventral hernias.

Early core-muscle activation has not shown an increased risk of fascial dehiscence or incisional hernia in the limited available studies [36]. Expert surveys also found little evidence for prolonged activity restriction [37,38]. These data do not define a phenotype-specific rehabilitation schedule, and the optimal timing and dose after complex reconstruction remain uncertain. Evidence for candidate interventions is in Table 3. 

**Table 3:** Evidence for candidate interventions: measured endpoints and relevance to phenotype-directed care.

Intervention	Source/Population	Design	Measured Endpoint	Main Result	Relevance to Personalised Care	Evidence Status
Open TAR	[14], n = 37	Retrospective paired CT	Muscle CSA	RA +16.1%; IO +16.5%; EO +9.5%	Documents morphometry; no function or metabolism	Direct, exploratory
Endoscopic vs. open anterior CS	[26], n = 163	Meta-analysis	Wound complications; recurrence	OR 0.27 for wound complications; recurrence similar	Technique comparison, not phenotype selection	Direct
BTA	[20,21,28,29,30,31,32]	Meta-analyses and cohorts	Muscle length; closure; recurrence	Elongation about 3.2–4.0 cm/side; closure improved	Selected by defect geometry and retraction	Direct; heterogeneous
BTA + PPP	[31], 20 studies	Meta-analysis	Closure; recurrence	Closure 0.94; recurrence 0.03	Anatomical adjunct for selected complex hernias	Direct; mostly observational
GLP-1 receptor agonist	[33], n = 70	Retrospective cohort	BMI; short-term recurrence	BMI −5.3 ± 3.5 kg/m^2^	Metabolic optimisation; no muscle endpoint	Direct cohort, uncontrolled
NMES	[34,35]; 6-study subset and 18-trial review	Meta-analyses of non-hernia and perioperative trials	Body fat; SMI; recovery	SMI +0.26 kg/m^2^; insufficient abdominal-surgery evidence	Candidate when active training is limited; not routine AAW care	Indirect
Early core training/activity	[36,37,38]	Systematic review and surveys	Safety of activity	No observed increase in dehiscence or hernia; evidence sparse	Supports individual progression; no fixed protocol	Direct; low certainty
Resistance exercise	[8]	Mechanistic review	Reinnervation and mass	Qualitative evidence	Candidate muscle-directed component	Indirect

BTA—botulinum toxin type A, CS—component separation, CSA—cross-sectional area, EO—external oblique, IO—internal oblique, NMES—neuromuscular electrical stimulation, PPP—progressive preoperative pneumoperitoneum, RA—rectus abdominis, SMI—skeletal muscle index, TAR—transversus abdominis release. Direct evidence was generated in hernia populations. Indirect evidence came from other surgical or muscle populations and cannot establish an AAW-specific treatment effect.

### 3.7. Integrative Model: The Vicious Cycle and Its Rupture Points

Direct data support individual parts of the proposed cycle, including associations between obesity and hernias, extracellular-matrix changes in hernia tissue, lateral muscle displacement, impaired function and postoperative CT morphometry [1,6,12,13,14,16,17,22]. The links from a preoperative muscle phenotype to a specific postoperative trajectory or recurrence remain hypothetical. Figure 1 distinguishes these levels of support with solid and dashed arrows.

The model identifies perioperative points for study rather than a treatment algorithm. Preoperative weight management may be used when clinically indicated [33]; BTA is selected by defect geometry and muscle retraction [20,21,28,29,30,31,32]; posterior reconstruction can preserve neurovascular supply while restoring midline anatomy [13,14,26,27]; and graded postoperative activity is consistent with the limited safety evidence [36,37,38]. Whether any of these measures change muscle attenuation, biomarkers or a proposed trajectory has not been shown.

Until AAW-specific thresholds and outcome associations are established, muscle phenotype should not independently determine operative timing or technique. Quantitative CT and functional measures can instead be collected prospectively alongside established clinical variables.

## 4. Discussion

The evidence permits three narrow conclusions. Chronic ventral hernias are associated with altered AAW geometry and reduced function [12,16,17]. Sarcopenia has an inconsistent association with outcomes in the available hernia cohorts [9,11]. Open TAR is followed by increased CT muscle area, but the functional and metabolic meaning of this change is unknown [14]. The literature does not yet show that obesity, metabolic syndrome and hernia form a causal triad within muscle tissue or that baseline phenotype predicts one of three postoperative trajectories.

The relevance to personalised medicine lies in measurable heterogeneity. Two patients with the same defect width may differ in muscle area, attenuation, intermuscular fat, denervation, glycaemic control and physical reserve. A research framework that records these variables can test whether they alter responses to weight optimisation, BTA, reconstruction or rehabilitation. It should not be presented as a validated staging system.

### 4.1. Proposed Framework for Personalised Assessment

CT measurement. On an abdominal CT scan obtained for clinical reasons, record the muscle cross-sectional area, mean attenuation and intermuscular fat at a prespecified L3–L4 level. Report continuous values; no AAW-specific diagnostic cut-off is available.

Clinical and functional context. Interpret CT together with BMI or waist measures, diabetes control, hernia duration, defect geometry, prior incisions and a reproducible strength or performance measure where feasible [12,17].

Modifiable domain. Use weight management for established metabolic indications, BTA for anatomical retraction or loss of domain, and supervised resistance exercise with nutritional assessment for low strength or mass. NMES remains a candidate when active exercise is not feasible [20,21,33,34,35].

Biomarkers. MMP/TIMP, collagen-turnover and oxidative-stress markers should be measured only in a research protocol until thresholds and outcome associations are established.

Longitudinal assessment. Repeat the same imaging and functional measures at defined intervals and relate changes to complications, recurrence and patient-reported function. This design tests the framework without assuming that CT area equals recovered muscle.

This approach uses data already available in many complex-hernia pathways and defines what must be validated before clinical use. The proposed measures may organise data collection and trial stratification; they should not replace established anatomical assessment or current indications for treatment.

### 4.2. Gaps in the Evidence Base

Direct data on myosteatosis of the AAW in elective ventral-hernia cohorts are absent. A PubMed search on 30 April 2026 for “myosteatosis AND abdominal wall hernia” identified one relevant study [10], conducted in kidney-transplant recipients; myosteatosis and sarcopenia were not significant predictors of incisional hernias in its CT subgroup. This result cannot provide reference values for elective ventral hernias.

No study has measured mitochondrial function in ventral-hernia AAW muscle. Human fibre-type morphometry is sparse, serial biopsies before and after reconstruction have not been reported, and no randomised trial has compared prehabilitation regimens using muscle imaging or biomarkers as the primary endpoint.

Abdominal CT is commonly available before complex reconstruction [9]. A reproducible research protocol should specify contrast phase, anatomical level, muscle boundaries and segmentation method, and should report the cross-sectional area, mean attenuation and intermuscular fat separately. Serial studies should use the same acquisition and analysis method. Published thresholds from other anatomical levels and populations cannot be transferred to the AAW [7,10]; sex- and protocol-specific reference values must be derived in ventral-hernia cohorts before categorical classification is attempted. Table 4 links the principal gaps to study designs and decision-relevant endpoints.

### 4.3. Limitations

This is a structured narrative review. Two authors screened and selected sources; no protocol was registered, no formal record of retrieved, duplicate or excluded reports was preserved, and no PRISMA or PRISMA-ScR flow diagram can be reconstructed. A formal risk-of-bias assessment was not performed. The 38 publications constitute a selected body of evidence rather than a systematic corpus. Direct ventral-hernia evidence is sparse and consists largely of small retrospective or imaging series; several mechanistic claims rely on inguinal fascia, transplantation, geriatric or oncological cohorts, general muscle biology and animal models. No prospective study links a preoperative AAW phenotype to a postoperative remodelling trajectory. The CT cut-offs, functional thresholds and biomarker values have not been validated for treatment selection. The three-trajectory model and the proposed assessment framework should therefore guide research design, not current practice in isolation. The English-language restriction may have introduced selection bias, and relevant work may have been missed.

## 5. Conclusions

Direct studies show that a chronic ventral hernia is accompanied by altered AAW geometry and function and that muscle area changes after reconstruction. They do not show that a preoperative metabolic phenotype causes recurrence or predicts one of the three proposed postoperative trajectories.

Routine CT offers a practical way to record muscle area, attenuation and intermuscular fat, but AAW-specific reference values and outcome-linked cut-offs are absent. Candidate circulating and tissue biomarkers remain research measures.

Prospective studies should combine standardised CT, functional testing and clinical variables, then relate serial changes to complications, recurrence and patient-reported function. Until those associations are demonstrated, weight management, BTA, operative technique and rehabilitation should be selected according to established clinical and anatomical indications rather than an unvalidated muscle phenotype.

## Figures and Tables

**Table 1:** Candidate muscle phenotypes in ventral hernias: operational features, evidence and limits of clinical use.

Parameter	Myosteatosis	Sarcopenia	Sarcopenic Obesity
Operational pattern	Lower attenuation and/or increased IMAT; CSA may be preserved	Reduced CSA and function; attenuation may be preserved	Low CSA with low attenuation or excess IMAT
Common clinical context	Obesity, insulin resistance, T2DM	Ageing, catabolic disease, malnutrition or denervation	Obesity with low muscle reserve
Main concern	Reduced muscle quality and endurance	Reduced force and reserve	Combined quality and quantity deficit
CT status in the AAW	No validated cut-off	No validated AAW index or cut-off	No validated definition
Direct hernia evidence	Absent for elective ventral hernia; negative transplant subgroup [10]	Limited and inconsistent [9,11]	Prevalence and outcomes unknown

AAW—anterior abdominal wall, CSA—cross-sectional area, CT—computed tomography, IMAT—intermuscular adipose tissue, T2DM—type 2 diabetes mellitus. All three patterns remain research phenotypes and should not determine treatment alone; candidate modifiable domains are discussed in Section 4.1.

**Table 2:** Candidate markers of extracellular-matrix and muscle biology: source, evidence and current role.

Marker or Group	Material/Source	Reported Finding	Direct Evidence in Ventral AAW Muscle	Current Role	Source(s)
MMP-2/TIMP-2	Plasma and transversalis fascia	MMP-2 increased and TIMP-2 decreased in inguinal hernia studies	No	Research only; no validated cut-off	[22,23]
Collagen I/III ratio	Skin, fascia and muscle stroma	Lower ratio reported in hernia biology	Partial; mainly fascia/stroma	Research only	[1,6]
Picrosirius red-stained fibre area	Transversalis fascia	Association with major complications after subcostal laparotomy	No	Indirect prognostic evidence	[4]
P1NP/Pro-C3	Serum	Not established in ventral hernia	No	Candidate longitudinal endpoints	[1]
TNF-α/IL-6/TGF-β	Visceral adipose tissue and general muscle studies	Catabolic and profibrotic mechanisms	No	Mechanistic context only	[5,6]
ESR1 pathway	Lower-abdominal fibroblasts in a mouse model	ESR1 ablation reduced fibrosis and herniation	Preclinical only	No human treatment inference	[24]
8-OHdG/4-HNE/MDA	Muscle tissue	Not measured in hernia-carrier AAWs	No	Proposed trial endpoints	—
Mitochondrial function	Muscle biopsy	Not measured in hernia-carrier AAWs	No	Proposed trial endpoint	—

AAW—anterior abdominal wall, ESR—oestrogen receptor alpha, MDA—malondialdehyde, MMP—matrix metalloproteinase, P1NP—N-terminal propeptide of type I procollagen, Pro-C3—C-terminal propeptide of type III procollagen, TIMP—tissue inhibitor of metalloproteinase. No marker in this table has a validated threshold for treatment selection in ventral hernias.

**Table 4:** Research priorities for validation of personalised AAW assessment.

Knowledge Gap	Current Evidence	Missing Information	Proposed Population	Primary Endpoint	Design	Decision to Be Tested
AAW CT reference values	Non-hernia CT studies [7,10]	Protocol- and sex-specific distributions	Consecutive elective VHR cohort	CSA, attenuation and IMAT	Multicentre cross-sectional study	Whether a reproducible phenotype can be defined
CT phenotype and outcomes	Inconsistent sarcopenia data [9,11]	Prospective outcome association	Adults undergoing VHR	Complications, recurrence and function	Prospective validation cohort	Whether CT adds information beyond clinical variables
Human muscle biology	Animal and general-muscle evidence [5,15]	Human fibre type and mitochondrial data	Patients undergoing planned AAW biopsy	Fibre morphometry and respiration	Biopsy substudy	Whether proposed mechanisms occur in human AAWs
Post-repair trajectory	Single-time-point morphometry [14]	Linked structural, functional and metabolic change	TAR and other reconstruction cohorts	Change in CT, strength and biomarkers	Longitudinal study at baseline, 6 and 12 months	Whether distinct trajectories exist
Muscle-directed prehabilitation	Indirect NMES and exercise evidence [8,34,35]	AAW-specific treatment effect	Patients with low muscle reserve	Strength, CT phenotype and recovery	Randomised controlled trial	Whether intervention modifies recovery
Biomarker-guided care	Fascial and inguinal-hernia evidence [1,22,23]	AAW thresholds and incremental value	Biobanked VHR cohort	MMP/TIMP and collagen markers	Prospective biomarker study	Whether biomarkers improve prediction

AAW—anterior abdominal wall, CSA—cross-sectional area, CT—computed tomography, IMAT—intermuscular adipose tissue, MMP—matrix metalloproteinase, TAR—transversus abdominis release, TIMP—tissue inhibitor of metalloproteinase, VHR—ventral hernia repair. Sample sizes in this agenda require formal calculation for each protocol. Multicentre recruitment and standardised imaging would be needed for external validation.

## Data Availability

No new data were created or analysed. All evidence discussed is available in the cited publications.

## Supplementary Materials

The following supporting information can be downloaded at https://www.mdpi.com/article/10.3390/jpm16080419/s1, Syntax was adapted to each database; the complete strings are provided in Supplementary Table S1.
