# Supplementary material for: Metabolism, Morphology and Function of the Anterior Abdominal Wall Musculature in Ventral Hernias and After Repair: A Narrative Review and Research Agenda
Source: J Pers Med. 2026 Aug 6;16(8):419. doi: 10.3390/jpm16080419 (PMC13514369; doi:10.3390/jpm16080419)
Supplement: Supplementary file 1 [file jpm-16-00419-s001.zip › jpm-4404072-supplementary.pdf]

Supplementary Table S1

1. Concept blocks

Concept block	Terms (as listed in Section 2, Methods)
Block A (Population / condition)	ventral hernia; incisional hernia; abdominal wall; abdominal wall reconstruction
Block B (Muscle phenotype)	sarcopenia; myosteatosis; muscle atrophy; muscle fibrosis; muscle remodeling
Block C (Metabolic / molecular drivers)	obesity; metabolic syndrome; collagen metabolism; matrix metalloproteinase; reperfusion injury
Block D (Interventions)	botulinum toxin; component separation; transversus abdominis release; GLP-1 receptor agonist; neuromuscular electrical stimulation

Combination logic: `Block A AND (Block B OR Block C OR Block D)` — i.e., every record had to contain at least one hernia/abdominal-wall term, intersected with at least one muscle-phenotype, metabolic, or intervention term. Terms within each block were combined with OR; blocks were combined with AND.

2. Search strings by database

2.1. PubMed / MEDLINE

```
("ventral hernia"[tiab] OR "incisional hernia"[tiab] OR "Hernia, Ventral"[Mesh]
  OR "abdominal wall"[tiab] OR "abdominal wall reconstruction"[tiab])
AND
("sarcopenia"[tiab] OR "Sarcopenia"[Mesh] OR "myosteatosis"[tiab]
  OR "muscle atrophy"[tiab] OR "Muscular Atrophy"[Mesh] OR "muscle fibrosis"[tiab]
  OR "muscle remodeling"[tiab] OR "obesity"[tiab] OR "Obesity"[Mesh]
  OR "metabolic syndrome"[tiab] OR "Metabolic Syndrome"[Mesh]
  OR "collagen metabolism"[tiab] OR "matrix metalloproteinase"[tiab]
  OR "Matrix Metalloproteinases"[Mesh] OR "reperfusion injury"[tiab]
  OR "Reperfusion Injury"[Mesh] OR "botulinum toxin"[tiab] OR "Botulinum Toxins"[Mesh]
  OR "component separation"[tiab] OR "transversus abdominis release"[tiab]
  OR "GLP-1 receptor agonist"[tiab] OR "Glucagon-Like Peptide-1 Receptor"[Mesh]
  OR "neuromuscular electrical stimulation"[tiab] OR "Electric Stimulation Therapy"[Mesh])
```

Filters applied: Language: English; Publication date: 01/01/2010–04/30/2026

2.2 Scopus

```
TITLE-ABS-KEY(
  ( "ventral hernia"  OR  "incisional hernia"  OR  "abdominal wall"
    OR  "abdominal wall reconstruction" )
```

```
  AND
  ( "sarcopenia"  OR  "myosteatosis"  OR  "muscle atrophy"  OR  "muscle fibrosis"
    OR  "muscle remodeling"  OR  "obesity"  OR  "metabolic syndrome"
    OR  "collagen metabolism"  OR  "matrix metalloproteinase"  OR  "reperfusion injury"
    OR  "botulinum toxin"  OR  "component separation"  OR  "transversus abdominis release"
    OR  "GLP-1 receptor agonist"  OR  "neuromuscular electrical stimulation" )
)
AND PUBYEAR > 2009 AND PUBYEAR < 2027
AND LANGUAGE ( english )
```

Filters applied: Language: English; Publication years: 2010–2026.

2.3 Web of Science (Core Collection)

```
TS=(
  ( "ventral hernia"  OR  "incisional hernia"  OR  "abdominal wall"
    OR  "abdominal wall reconstruction" )
  AND
  ( "sarcopenia"  OR  "myosteatosis"  OR  "muscle atrophy"  OR  "muscle fibrosis"
    OR  "muscle remodeling"  OR  "obesity"  OR  "metabolic syndrome"
    OR  "collagen metabolism"  OR  "matrix metalloproteinase"  OR  "reperfusion injury"
    OR  "botulinum toxin"  OR  "component separation"  OR  "transversus abdominis release"
    OR  "GLP-1 receptor agonist"  OR  "neuromuscular electrical stimulation" )
)
```

Filters applied: Refined by: Language = English; Timespan = 2010-01-01 to 2026-04-30.
